# Phase 2 study of TAS-117, an allosteric akt inhibitor in advanced solid tumors harboring phosphatidylinositol 3-kinase/v-akt murine thymoma viral oncogene homolog gene mutations

**DOI:** 10.1007/s10637-021-01085-7

**Published:** 2021-03-15

**Authors:** Jii Bum Lee, Minkyu Jung, Seung Hoon Beom, Gun Min Kim, Hye Ryun Kim, Hye Jin Choi, Joo Hyuk Sohn, Joong Bae Ahn, Sun Young Rha, Hyun Cheol Chung

**Affiliations:** 1 Division of Medical Oncology, Department of Internal Medicine, Yonsei Cancer Center, Yonsei University College of Medicine, 50-1 Yonsei-ro, Seodaemun-gu, Seoul, Korea; 2 Song-Dang Institute for Cancer Research, Yonsei University College of Medicine, Seoul, Korea; 3 Brain Korea 21 Plus Project for Medical Sciences, Yonsei University College of Medicine, Seoul, South Korea

**Keywords:** TAS-117, PI3K/Akt mutations, Solid tumors, Basket trial

## Abstract

TAS-117 is a potent and selective allosteric pan-v-akt murine thymoma viral oncogene homolog (Akt) inhibitor. We conducted a single-arm single-center phase 2 study of TAS-117 in heavily treated patients with tumors refractory to systemic chemotherapy and harboring phosphatidylinositol 3-kinase (PI3K)/Akt mutations. Patients with gastrointestinal (GI) cancers were orally administered 16 mg TAS-117 daily, and those with non-GI tumors were administered 24 mg on a 4 days on/3 days off schedule. The primary endpoint was overall response rate (ORR). Secondary endpoints included disease control rate (DCR), progression-free survival (PFS), overall survival (OS), PFS ratio, safety, and tolerability. Thirteen patients were enrolled: eight with non-GI (breast, ovarian, endometrial, and non-small cell lung) and five with GI (colon, rectal, gastric, and gallbladder) cancers. Ten patients were treated with TAS-117 after ≥ 4 lines of therapy. Twelve patients showed PIK3 catalytic subunit alpha (*PIK3CA*) mutations; one harbored an Akt1^E17K^ mutation. The median treatment duration was 1.4 months; the median number of treatment cycles was 2. The ORR was 8 %, and DCR was 23 %. The median PFS and OS were 1.4 and 4.8 months, respectively. Grade 3–4 treatment-related adverse events were anorexia (grade 3, 8 %) and hyperglycemia (grade 3, 8 %; grade 4, 8 %).Grade 3–4 treatment-related adverse events occurred in 27 % of grade 3 anorexia (9 %) and hyperglycemia (grade 3, 8 %; grade 4, 9\%). TAS-117 showed limited antitumor activity and manageable toxicity. Clinical efficacy was observed in patients with ovarian cancer harboring *PIK3CA* E545K mutations and in patients with breast cancer harboring *PIK3CA* H1047R and Akt1^E17K^ mutations.

Trial registration: This study was retrospectively registered with ClinicalTrial.gov (NCT03017521 on January 11, 2017).

**Supplementary Information:**

The online version contains supplementary material available at 10.1007/s10637-021-01085-7.

## Background

The phosphatidylinositol 3-kinase (PI3K)/v-akt murine thymoma viral oncogene homolog (Akt)/mammalian target of rapamycin (mTOR) signaling pathway plays a role in cell proliferation, cell cycle, apoptosis, and cancer cell metabolism [1]. The dysregulation of this pathway has been associated with various types of cancer [2]. Therefore, inhibiting the PI3K/Akt/mTOR pathway using targeted agents may clinically benefit patients harboring mutations in genes associated with this pathway.

Over the past few years, more than 40 novel compounds targeting the PI3K/Akt/mTOR pathway have been developed [3]. However, only few agents such as mTOR inhibitors (temsirolimus and everolimus) and PI3K inhibitors (idelalisib, a δ-specific PI3K inhibitor; copanlisib, a PI3K inhibitor predominantly active against PI3K-α and PI3K-δ; and alpelisib, an α-specific PI3K inhibitor) have been approved by the United States Food and Drug Administration (FDA) [4–9]. Although FDA-approved, allosteric mTOR inhibitors such as temsirolimus and everolimus have shown low objective response rates when administered as monotherapies [4, 6]. With the exception of copanlisib, pan-PI3K inhibitors show intolerable toxicities owing to their broad spectrum molecular activities [10]. Other agents have failed because of poor efficacy, high toxicity, or a lack of reliable predictive biomarkers [11].

Several preclinical studies using cell lines and xenografts have shown that targeting the downstream Akt pathway reduces cell proliferation in multiple tumor cell lines [12]. Akt activation results from abnormalities in upstream regulators including (1) upstream activation or gene amplification of receptor tyrosine kinases, (2) amplification of or mutations in the PI3K catalytic subunit alpha (*PIK3CA*) gene encoding the p110α catalytic subunit of PI3K, or (3) gene silencing of phosphatase and tensin homolog (PTEN), a tumor suppressor gene that negatively regulates the PI3K pathway [13].

TAS-117 (Taiho Pharmaceutical Co., Ltd., Tokyo, Japan) is a highly potent and selective oral allosteric Akt inhibitor that shows high affinity for all three isoforms (Akt1, 2, and 3) [14]. It inhibits the proliferation of human cancer cell lines *in vitro*, including breast, endometrial, lung, and ovarian cancer cells [15]. Furthermore, tumor cell lines sensitive to TAS-117 include those with Akt2 and human epidermal growth factor type 2 (HER2) gene amplification, PI3K mutations, and PTEN loss. In a nude mouse xenograft model, the daily administration of TAS-117 caused significant dose-dependent, antitumor effects. A phase 1, all-comers study of TAS-117 including 60 patients with evaluable advanced solid tumors showed promising objective responses, especially in patients with ovarian cancer. In addition, TAS-117 showed a manageable safety profile across patients with all cancer types. The recommended phase 2 doses (RP2Ds) were 16 mg daily and 24 mg, 4 days on/3 days off, because of the dose-limiting toxicity of maculopapular rash [14]. Therefore, we conducted a phase 2 study of TAS-117 in patients with advanced solid tumors harboring PI3K/Akt gene aberrations as part of the Korea-Biomarker-driven multi-arm drug-screening, knowledge and evidence-generating targeted trial (K-BASKET trial). In this study, we report the efficacy and safety of TAS-117 in multiple cancer types that are refractory to standard treatments.

## Methods

### Study design and patients

This phase 2 trial of TAS-117 is part of the K-BASKET trial conducted at the Yonsei Cancer Center, Korea. The trial was registered at ClinicalTrials.gov (NCT03017521). Eligibility criteria included patients with histologically or cytologically confirmed recurrent or advanced solid cancers with PI3K/Akt gene aberrations identified via next-generation sequencing (NGS), with one of the following mutations: (1) *PIK3CA* mutations in E542X, E545K, Q546X, Q546X, or H1047X; (2) Akt^E17K^; or (3) Akt1/Akt2 amplifications. Other eligibility criteria included previous standard treatment failure; at least one measurable lesion according to the Response Evaluation Criteria in Solid Tumors (RECIST) version 1.1 criteria [16]; Eastern Cooperative Oncology Group (ECOG) performance status score of 0 (fully active) to 1 (ambulatory and capable of self-care); ability to receive medication orally without feeding tube; life expectancy ≥ 60 days; adequate hematological, hepatic, and renal function; fasting serum glucose ≤ 160 mg/dL; glycosylated hemoglobin ≤ 8.0 %; low-density lipoproteins ≤ 190 mg/dL; and triglycerides ≤ 300 mg/dL. The key exclusion criteria included prior treatments targeting PI3K/Akt gene aberrations, retinopathy requiring treatment, and concurrent treatment requiring steroids.

All authors followed Good Clinical Practice, and the study was conducted according to the principles of the Declaration of Helsinki. All patients enrolled provided written informed consent. The trial was conducted in accordance with the CONsolidated Standards of Reporting Trial (CONSORT), and the protocol was approved by the Institutional Review Board of Severance Hospital (IRB 4-2016-0743).

### Study treatment

 The study was conducted over 21-day treatment cycles. The doses were based on results from the phase 1 trial, and the RP2D was calculated according to tumor types (gastrointestinal [GI] or non-GI tumors). Patients with GI tumors received 16 mg of TAS-117 daily under fasting conditions (1 h before or 2 h after a meal), and patients with non-GI cancers received 24 mg, 4 days on/3 days off. Dose interruptions, up to 21 days, and dose reductions, to 12 mg (level − 1) and 8 mg (level − 2) for GI cancers and 20 mg (level − 1) and 16 mg (level − 2) non-GI cancers, were allowed. Treatment was continued until disease progression, the occurrence of an unacceptable adverse event (AE), drug interruption for > 21 days, > 2 dose reductions, or withdrawal.

### Assessments

Disease status was assessed using contrast-enhanced computed tomography at the baseline, every 6 weeks, and at progression according to RECIST v1.1. Tumor markers such as carcinoembryonic antigen, carbohydrate antigen 19 − 9, cancer antigen-125, and cancer antigen 72 − 4 were assessed according to tumor type and represented as the percent change from the baseline. AEs and treatment-related AEs were evaluated throughout treatment and 30 days after the end of treatment using the National Cancer Institute Common Terminology Criteria for Adverse Events v4.3.

NGS was performed using archival tumor specimens obtained prior to TAS-117 treatment. An in-house panel (Cancer Master), a pan-cancer NGS platform that includes 524 genes and was developed in the Department of Pathology and Song-Dang Institute for Cancer Research, was used to assess co-existing mutations, including single nucleotide variants, insertion–deletions, and copy number variants.

### Statistics

To apply a one-sided significance level of 5 % and 90 % power, 25 patients were required to reject the null hypothesis that the upper bound of the 95 % confidence interval (CI) of the overall response rate (ORR) was < 25 %. Assuming a 20 % drop out rate, the final sample size was 30 patients for both GI cancers and non-GI cancers in a 24-month enrolment period.

All patients receiving ≥ 1 dose of TAS-117 were evaluated for endpoints related to efficacy and AEs. The primary endpoint was ORR, according to RECIST v1.1. The secondary endpoints included disease control rate (DCR), progression-free survival (PFS), overall survival (OS), PFS ratio, and the safety and tolerability of TAS-117. Exploratory endpoints included clinical responses to TAS-117 according to PI3K/Akt aberration subtypes and the identification of co-existing mutations associated with sensitization or resistance to PI3K/Akt aberrations.

The ORR was defined as the proportion of patients with the best overall complete or partial response. The Kaplan–Meier method was used to estimate median PFS and OS. Safety and tolerability were analyzed using descriptive statistics. Analyses were conducted using SPSS statistical software v25 (IBM Corp., Armonk, NY, USA) and GraphPad Prism 8 (GraphPad Software, Inc., San Diego, CA, USA).

## Results

### Patients

Between November 21, 2017 and June 27, 2019, 13 patients with advanced solid tumors harboring PI3K/Akt gene aberrations were screened and enrolled. The baseline characteristics are listed in Table 1. The median age was 53 years (range, 34–71), and 12 patients (92 %) were female. The ECOG performance status was 0 for eight patients (62 %).

**Table 1:** Baseline characteristics of patients

Median age (range)	53 (34–71)		
Sex			
Female	12 (92 %)		
Male	1 (8 %)		
ECOG*			
0	8 (62 %)		
1	5 (39 %)		
Cancer			
Breast cancer	4 (31 %)		
Ovarian cancer	2 (15 %)		
Endometrial cancer	1 (8 %)		
Colon cancer	2 (15 %)		
Rectal cancer	1 (8 %)		
Gastric cancer	1 (8 %)		
Gallbladder cancer	1 (8 %)		
NSCLC	1 (8 %)		
Line of treatment for TAS-117			
2	1 (8 %)		
3	2 (15 %)		
4	6 (46 %)		
5	2 (15 %)		
6	1 (8 %)		
9	1 (8 %)		
Royal Marsden Score			
0	1 (8 %)		
1	5 (38 %)		
2	6 (46 %)		
3	1 (8 %)		
GRIm-Score			
0	6 (46 %)		
1	5 (38 %)		
2	1 (8 %)		
3	1 (8 %)		
Number of metastatic organs			
1	1 (8 %)		
2	6 (46 %)		
3	4 (31 %)		
5	2 (15 %)		

Abbreviations: ECOG, Eastern Cooperative Oncology Group; GRIm-Score, Gustave Roussy Immune Score

^*^An ECOG performance status score 0 means that the patient is fully active and 1 means that the patient is restricted in physically strenuous activity but ambulatory

Eight patients with non-GI cancers were enrolled, including breast (n = 4, 31 %), ovarian (n = 2, 15 %), endometrial (n = 1, 8 %), and non-small cell lung cancer (NSCLC; n = 1, 8 %). In addition, five patients with GI cancers, including colon (n = 2, 15 %), rectal (n = 1, 8 %), gastric (n = 1, 8 %), and gallbladder cancer (n = 1, 8 %), were enrolled. With the exception of three patients who received TAS-117 as second- or third-line treatment, most of the patients (n = 10, 77 %) treated with TAS-117 had already received more than three lines of treatment. In addition, 46 % (n = 6) and 84 % (n = 11) of patients had Royal Marsden Scores (RMS) and Gustave Roussy Immune Scores (GRIm-Scores) of 0 and 1, respectively. Most patients had two or more metastatic organs (n = 12, 92 %). Twelve patients showed mutations in *PIK3CA*: E542K (n = 2, 15 %), E545A (n = 1, 8 %), E545K (n = 4, 31 %), H1047R (n = 4, 31 %), and Q546K (n = 1, 8 %), and one patient harbored Akt1^E17K^ mutations (Supplementary Table S1).

### Antitumor activity

**Fig. 1:**
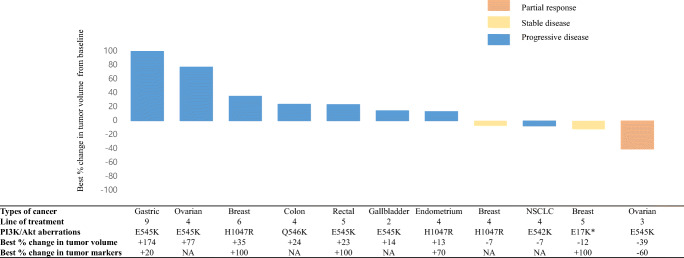
Waterfall plot depicting best percent changes in target tumor burden. All patients harbored PI3K aberrations, except for patients with breast cancer harboring AKT E17K* mutations. Two patients with clinical progression were excluded from this graph. Seven subjects did not undergo subsequent follow-up of tumor markers. Abbreviations: NSCLC, non-small cell lung cancer; NGG, next-generation sequencing; PI3K/Akt, phosphatidylinositol 3-kinase/protein kinase B; NA, not applicable (no follow-up of tumor markers for assessment)

At the end of the data collection period on February 19, 2020, the median follow-up duration was 6.6 months (range, 1–18.1 months). The median duration of treatment was 1.4 months (range, 0.4–3.2 months), and the median number of treatment cycles was 2 (range, 1–5; Supplementary Table S2). Of the 13 patients, none showed a complete response, one patient with ovarian cancer showed a confirmed partial response, and two patients with breast cancer had stable disease **(**Fig. 1**)**. Eleven patients were assessed via radiology, but two patients did not have radiological assessments because of rapid clinical deterioration.

**Fig. 2:**
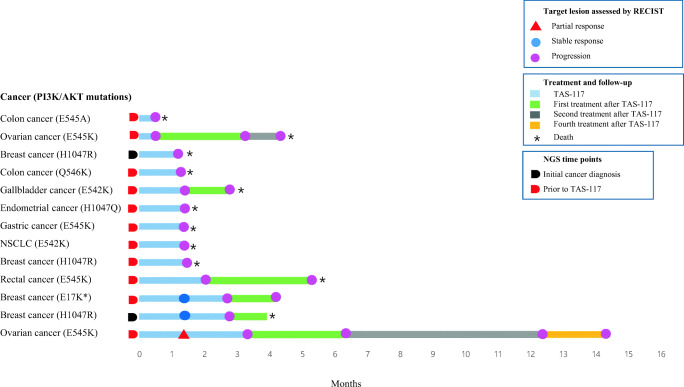
Swimmer plot depicting progression-free survival (PFS). All patients harbored PI3K aberrations, except for one patient with breast cancer harboring Akt E17K*. Abbreviations: RECIST, response evaluation criteria in solid tumors; NSCLC, non-small cell lung cancer; NGS, next-generation sequencing

The ORR and DCR of TAS-117 were 8 % (n = 1) and 23 % (n = 3), respectively (Supplementary Table S3). All responses were achieved in patients with non-GI tumors, and the median time to response was 6 weeks (Supplementary Figure S1A). One patient with metastatic ovarian cancer harboring PI3Kα E545K mutations showed a 39 % tumor reduction at 6 weeks (data not shown). However, this decrease in tumor burden was not maintained over subsequent assessments, and the patient showed progression in non-target lesions and an increase in tumor markers at 12 and 18 weeks (Supplementary Figure S1B). The two patients with breast cancer who achieved stable disease condition (n = 2, 15 %) and harbored Akt1^E17K^ and PI3Kα H1047R mutations showed disease progression at 12 weeks, associated with an increase in non-target lesions. Other patients with breast cancer (n = 2, 15 %), ovarian cancer (n = 1, 8 %), GI cancer (n = 5, 38 %), and NSCLC (n = 1, 8 %) showed progression at the first response evaluation, as depicted in both the spider and swimmer plot (Supplementary Figure S1 and Fig. 2, respectively). In eight patients expressing tumor markers, there was an increase in the percentage of tumor markers at 6 weeks in six patients, which did not correlate with radiological responses (Supplementary Figure S1B). A summary of the best responses according to PI3K/Akt aberrations is shown in Supplementary Table S5.

**Fig. 3:**
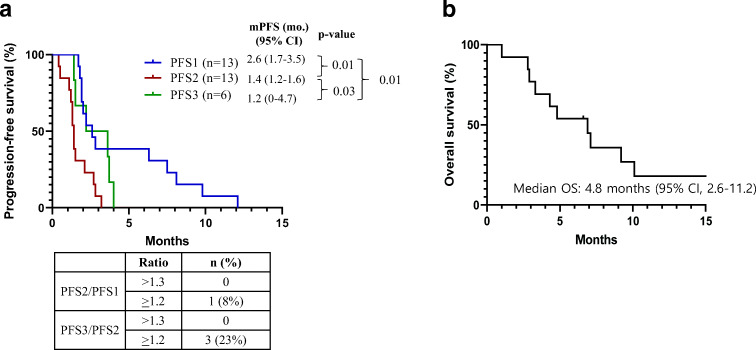
Analysis of progression-free survival (PFS) and overall survival (OS). (a) PFS 1 (prior to TAS-117), PFS 2 (TAS-117), and PFS 3 (after TAS − 117). (b) OS. Abbreviations: mPFS, median progression-free survival; OS, overall survival, CI, confidence interval; mo., months

The median PFS was 1.4 months (95 % CI: 1.2–1.6 months) for TAS-117 treatment (Fig. 3a, Supplementary Figure S2). PFS values prior to (PFS 1) and after (PFS 3) TAS-117 treatment were 2.6 (95 % CI: 1.7–3.5 months) and 1.2 months (95 % CI: 0–4.7 months), respectively. PFS2/1 ratio > 1.3 and PFS3/2 ratio > 1.3 were both 0 %, and PFS2/1 ≥ 1.2 ratio and PFS3/2 ≥ 1.2 ratio were 8 % and 23 %, respectively [17]. The median OS was 4.8 months (95 % CI: 2.6–11.2 months; Fig. 3b). A univariate analysis of treatment line (< 4 vs. ≥ 4), RMS (0 or 1 vs. ≥ 2), GRIm-Score (0 or 1 vs. ≥ 2), and number of metastatic lesions (< 2 vs. ≥ 2) showed that there was no statistical significance in either PFS or OS (Supplementary Table S6).

Nine patients had disease progression, two experienced adverse events, one withdrew from the study, and one discontinued treatment owing to the physician’s decision. Of the 11 evaluable patients, most showed progression in previous target lesions (n = 9, 69 %), such as the lungs (n = 6, 55 %), peritoneal carcinomatosis (n = 5, 46 %), and liver (n = 4, 36 %; Supplementary Table S7). One patient developed a new site of metastasis in the liver (n = 1), and one patient developed new lesions in both the common bile duct and pancreas. The two patients without radiological assessments also showed clinical progression. A one-way analysis of variance (ANOVA) showed no statistical difference in progression between organs (p = 0.076; Supplementary Figure S3).

At progression, six patients (46 %) received subsequent treatment, including cytotoxic chemotherapy (n = 3, 23 %), immunotherapy (n = 2, 15 %), and targeted agents (n = 1, 8 %; Supplementary Table S2). At the time of analysis,11 patients had died, one patient was receiving best supportive care, and one patient was receiving subsequent chemotherapy **(**Fig. 2**)**.

### Co‐existing mutations

In this study, patients with PI3K/Akt aberrations also harbored diverse co-existing mutations (n = 22; Supplementary Fig. 4). The most common mutations identified were tumor protein 53 (TP53) mutations (n = 7), including missense mutations (n = 5), frameshift deletion (n = 1), and frameshift insertion (n = 1). There were no statistically significant correlations with regard to the site of biopsy (primary vs. metastatic), biopsy time period (initial cancer diagnosis vs. prior to TAS-117 treatment), response evaluation according RECIST criteria (partial response, PR; stable disease, SD; progressive disease, PD), or tumor type (Supplementary Fig. 5).

### Safety

AEs were evaluated in all 13 patients (Supplementary Table S8). Treatment-related AEs are listed in Table 2. Overall, 11 patients (85 %) experienced ≥ 1 treatment-related AEs, including hyperglycemia (all grades, n = 4, 36 %), skin rash (all grades, n = 4, 36 %), anorexia (all grades, n = 4, 36 %), nausea (all grades, n = 2, 15 %), and diarrhea (all grades, n = 2, 15 %). Notably, two patients (15 %) experienced grade 3 (n = 1, 8 %) and grade 4 hyperglycemia (n = 1, 8 %; Supplementary Table S9). Patients who were scheduled to receive dose reductions progressed radiologically and discontinued treatment. One patient experienced grade 3 anorexia (n = 1, 8 %), which was well managed with supportive care. The median dose intensity for TAS-117 was 100 % for both cohorts 1 and 2 (Supplementary Table S2, Figure S6). No patient death occurred as a result of treatment-related AEs.

**Table 2:** Incidence of treatment-related adverse events

Treatment-related adverse events	Grade 1	Grade 2	Grade 3	Grade 4	All grades
Any event	7 (54 %)	17 (100 %)	2 (15 %)	1 (8 %)	27 (100 %)
Anorexia	1 (8 %)	2 (15 %)	1 (8 %)	0 (0 %)	4 (36 %)
Constipation	0 (%)	1 (8 %)	0 (0 %)	0 (0 %)	1 (8 %)
Diarrhea	1 (8 %)	1 (8 %)	0 (0 %)	0 (0 %)	2 (15 %)
Nausea	1 (8 %)	1 (8 %)	0 (0 %)	0 (0 %)	2 (15 %)
Mucositis	1 (8 %)	0 (0 %)	0 (0 %)	0 (0 %)	1 (8 %)
Skin rash	2 (15 %)	2 (15 %)	0 (0 %)	0 (0 %)	4 (36 %)
Itching	0 (0 %)	1 (8 %)	0 (0 %)	0 (0 %)	1 (8 %)
Fatigue	0 (0 %)	1 (8 %)	0 (0 %)	0 (0 %)	1 (8 %)
Headache	0 (0 %)	1 (8 %)	0 (0 %)	0 (0 %)	1 (8 %)
Back pain	0 (0 %)	1 (8 %)	0 (0 %)	0 (0 %)	1 (8 %)
Shoulder pain	0 (0 %)	1 (8 %)	0 (0 %)	0 (0 %)	1 (8 %)
Dyspnea	0 (0 %)	1 (8 %)	0 (0 %)	0 (0 %)	1 (8 %)
Pneumonia	0 (0 %)	1 (8 %)	0 (0 %)	0 (0 %)	1 (8 %)
Pulmonary thromboembolism	0 (0 %)	1 (8 %)	0 (0 %)	0 (0 %)	1 (8 %)
Hyperglycemia	1 (8 %)	1 (8 %)	1 (8 %)	1 (8 %)	4 (36 %)
Neutropenia	0 (0 %)	1 (8 %)	0 (0 %)	0 (0 %)	1 (8 %)

NOTE. Adverse events were those with onset after enrolment to last follow-up after disease progression

Some of the percentages are rounded up or down and may not equal in sums.

## Discussion

Preliminary results of an all-comers phase 1 study of TAS-117 in 62 patients with advanced solid tumors showed that among 20 patients with ovarian clear cell carcinoma, five (25 %) experienced > 30 % tumor shrinkage and three (15 %) showed an ongoing response [14]. Similarly, our study showed that one of the four patients with ovarian cancer (25 %) achieved a partial response. This response, however, was not durable, and the patient showed disease progression at 18 weeks, which did not correlate with tumor markers. The other 12 patients, including those with SD, showed radiological disease progression. Ten patients experienced initial radiological progression at 6 weeks, and two patients showed clinical progression before completing the first treatment cycle. Overall, the short PFS and non-significant PFS ratios observed reflected the minimal benefits of administering TAS-117 as monotherapy. The main reasons for the recommendations by the independent data monitoring committee to terminate this study early and develop future combinations as earlier treatment lines were the (1) lack of durable response to TAS-117 in heavily treated patients with diverse cancer types who were refractory to standard treatments and (2) low accrual rate attributed to the low frequency of PI3K/Akt gene aberrations.

The lack of response to TAS-117 was attributed to several factors. First, Akt inhibition alone is insufficient in targeting the PI3K/Akt/mTOR pathway, as this shows various alterations [18]. Studies have shown intrinsic feedback regulation and acquired resistance mechanisms to PI3K inhibitors directly via canonical effectors of the pathway or parallel pathways that crosstalk with this signaling cascade [19]. To block other compensatory signaling pathways and increase antitumor activities, the administration of a combination of targeted treatments may be a feasible option. However, drugs co-targeting this pathway show similar toxicities, thus making the development of treatment combinations difficult [20]. For instance, buparlisib, a pan-class 1 PI3K inhibitor, shows high toxicity with adverse effects such as depression and anxiety, rendering its use inadequate in clinical settings [10]. Similarly, dual pan-PI3K and mTOR inhibitors have also shown high toxicity levels, and their development was discontinued [21].

Concurrent treatment with chemotherapy or immunotherapy without overlapping toxicities may optimize the inhibition of the PI3K/Akt/mTOR pathway [19]. Notably, PI3K and Akt inhibitor combinations have shown efficacy, especially in the treatment of breast cancer. Understanding the mechanisms linking estrogen receptor (ER) and the PI3K pathway has paved the way for combination treatment using fulvestrant (ER antagonist) and alpelisib, which shows synergistic antitumor activity in patients with *PIK3CA*-mutated, ER-positive, HER2-negative, advanced breast cancer unresponsive to previous estrogen therapy [9]. The loss of PTEN has also been associated with resistance to T-cell–mediated immunotherapy, and the combination of immunotherapy with PI3K/Akt/mTOR pathway inhibitors may be a promising treatment option [22]. A first-line triplet regimen comprising ipatasertib, an oral ATP-competitive Akt inhibitor, atezolizumab, a programmed death-ligand 1 (PD-L1), and a chemotherapy agent, is currently under investigation in metastatic triple-negative breast cancer [23]. Furthermore, first-line treatment with ipatasertib and abiraterone acetate improves radiographic PFS in metastatic castration-resistant prostate cancer with PTEN loss [24]. In this study, however, we were unable to identify *de novo* co-existing mutations correlated with responses to TAS-117.

Second, there are currently no standardized, reliable predictive biomarkers to select patients who can undergo treatment with agents targeting the PI3K/Akt/mTOR pathway. A previous study showed that *PIK3CA* gene mutations predict responses or prolonged PFS, but contradictory results were also observed [18]. Other clinical models have shown that cells with a loss of PTEN expression are more sensitive to Akt/PI3K inhibitors [25]. However, the definition and assessment of PTEN status via immunohistochemistry using different antibodies poses challenges for both patient selection and biomarker analysis [26]. Other preclinical data have shown that Akt^E17^ mutation may be a useful biomarker, but further proof-of-concept studies are required [27]. In preclinical settings, TAS-117 was sensitive to Akt2 and HER2 gene amplification, PI3K mutation, and PTEN loss. Nevertheless, none of the patients in our study showed concomitant Akt2 amplification and PTEN loss. One patient with breast cancer harboring the PI3KCA H1047R mutation also showed HER2 amplification but experienced no clinical response.

The mutation most commonly identified was a TP53 missense mutation, which was observed in five patients. Among the two patients with breast cancer who achieved SD, one harbored an Akt1^E17K^ mutation with a CDH1 frameshift deletion and another harbored a *PIK3CA* H1047R mutation with a KRAS missense mutation. The co-occurrence of TP53 and *PIK3CA* mutations is commonly observed in breast cancers. However, there is currently no evidence supporting the hypothesis that tumors enriched with these mutations benefit more from treatment with agents targeting the PI3K/Akt/mTOR pathway [28]. Similarly, CDH1 and *PIK3CA* have been commonly identified in invasive lobular breast carcinoma [29], but whether these concurrent mutations are sensitive to PI3K/Akt/mTOR agents remains to be confirmed. To our knowledge, there is no documented evidence showing that the combination of KRAS missense and *PIK3CA* H1047R mutations shows clinical relevance in breast cancer. Overall, there were no identifiable PI3K/Akt aberrations showing specific responses to TAS-117. A deeper genomic analysis will be prepared in a separate manuscript.

Finally, tumor types vary in terms of the duration and depth of responses to agents targeting the PI3K/Akt/mTOR pathway according to the degree of oncogenic addiction [30]. For some cancer types, a *PIK3CA* mutation may just be a subclonal driver mutation [31]. *PIK3CA* mutations have been commonly observed in many different solid tumors, and their responses vary across subtypes of PI3K [32]. Nonetheless, everolimus and alpelisib, in combination with other agents, are the only drugs to have shown clinical efficacy in breast cancer [6, 9], while temsirolimus is the only drug to have shown clinical efficacy in renal cell cancer [4]. Comparatively, other cancer types, such as rectal cancer, do not benefit from treatment with agents inhibiting this pathway [33]. Therefore, the proper selection of tumor types as well as driver mutations that oncogenically activate the PI3K/Akt/mTOR pathway is essential in achieving the full potential of PI3K/Akt/mTOR inhibitors.

In this single-center phase 2 trial of multiple solid tumors harboring PI3K/Akt aberrations, TAS-117 was well tolerated. AEs of special interest such as skin rash (all grades, n = 4, 36 %) and hyperglycemia (grade 3, n = 1, 8 %; grade 4, n = 1, 8 %) were well managed with dose interruption and supportive care. Patients who experienced hyperglycemia did not require dose reductions because they showed radiological progression before resuming treatment. The lack of AEs may be attributed to insufficient exposure to TAS-117. TAS-117 showed efficacy in one patient with ovarian cancer and disease control in two patients with breast cancer, suggesting specific histology and genotype preference; nonetheless, the short PFS suggests activation of collateral resistance pathways. However, our study did not require mandatory re-biopsy after treatment failure for the evaluation of adaptive resistance mechanisms.

## Conclusions

TAS-117 showed limited antitumor activity and a manageable toxicity profile in patients with diverse advanced solid tumors. Clinical efficacy was observed in patients with ovarian cancer (*PIK3CA* E545K mutation) and breast cancer (*PIK3CA* H1047R and Akt1^E17K^ mutations). *De novo* resistance to TAS-117 can be overcome by administering a combination treatment comprising chemotherapy, targeted therapy, and immunotherapy and including TAS-117 in earlier treatment lines in patients with breast and ovarian cancers to target *PIK3CA* E545K, H1047R, and Akt1^E17K^ mutations.

## Supplementary Information

ESM 1 (PDF 229 kb)

## Data Availability

The datasets used and/or analyzed during the current study are available from the corresponding author on reasonable request.
