# Supplementary material for: Phase 2 study of TAS-117, an allosteric akt inhibitor in advanced solid tumors harboring phosphatidylinositol 3-kinase/v-akt murine thymoma viral oncogene homolog gene mutations
Source: Invest New Drugs. 2021 Mar 15;39(5):1366–74. doi: 10.1007/s10637-021-01085-7 (PMC8426297; doi:10.1007/s10637-021-01085-7)
Supplement: Supplementary file 1 — (PDF 229 kb) [file 10637_2021_1085_MOESM1_ESM.pdf]

Investigational New Drugs

Phase 2 study of TAS-117 in advanced solid tumors harboring phosphatidylinositol 3-kinase/v-akt murine thymoma viral oncogene homolog gene aberrations

Jii Bum Lee[1,2], Minkyu Jung[1,2,3], Seung Hoon Beom[1,2], Gun Min Kim[1,2], Hye Ryun Kim[1,2], Hye Jin Choi[1,2], Joo Hyuk Sohn[1,2], Joong Bae Ahn[1,2,3], Sun Young Rha[1,2,3], Hyun Cheol Chung[1,2]

[1]*Division of Medical Oncology, Department of Internal Medicine, Yonsei Cancer Center, Yonsei University College of Medicine, Seoul, Korea*

[2]*Song-Dang Institute for Cancer Research, Yonsei University College of Medicine, Seoul, Korea*

[3]*Brain Korea 21 Plus Project for Medical Sciences, Yonsei University College of Medicine, Seoul, South Korea*

Correspondence: Hyun Cheol Chung, MD, PhD

Division of Medical Oncology, Department of Internal Medicine, Yonsei University College of Medicine, 50-1 Yonsei-ro, Seodaemun-gu, Seoul 03722, Korea

Tel.: 82-2-2228-8132 Fax: 82-2-393-3652 E-mail: unchung8@yuhs.ac

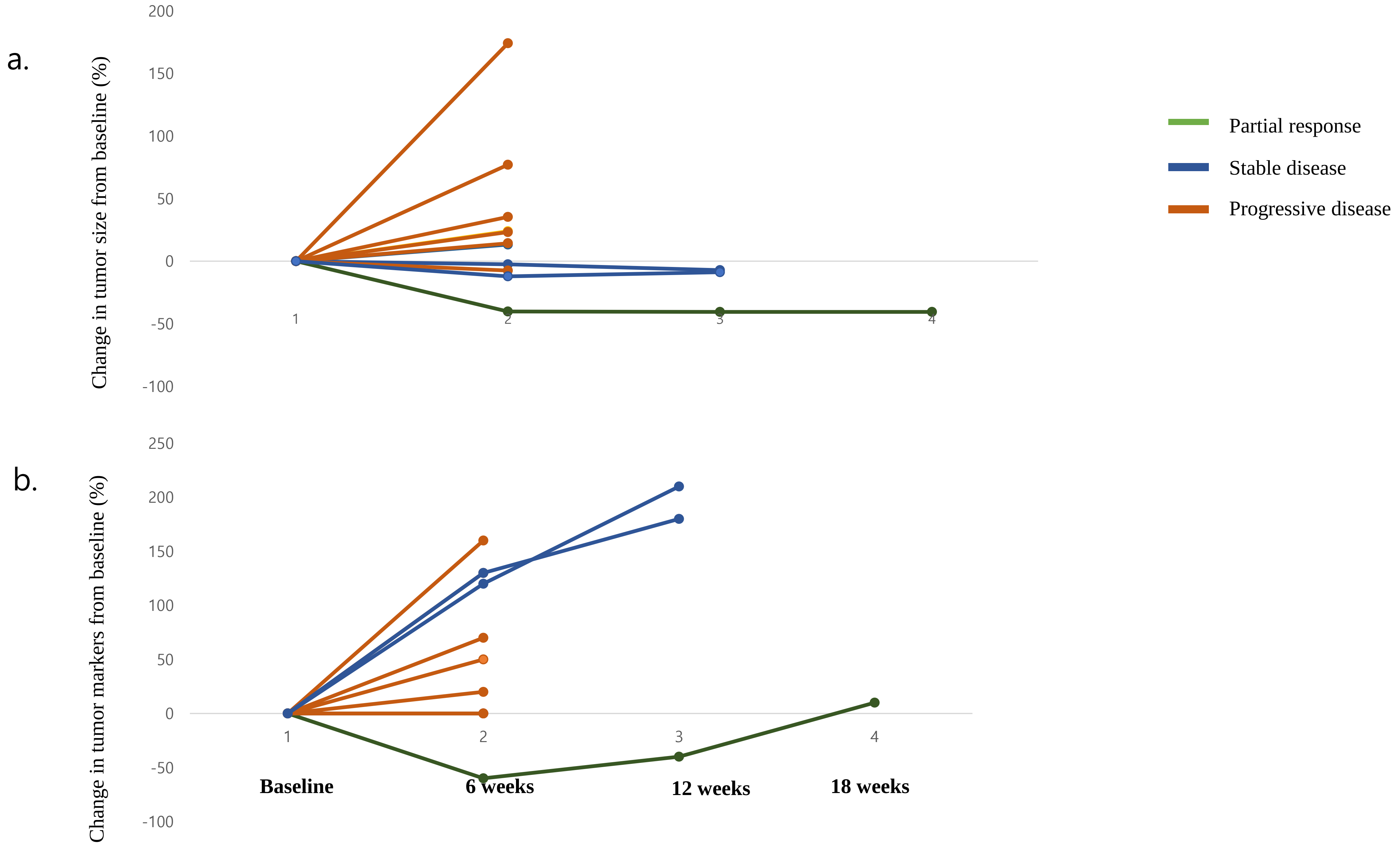

Fig. S1 Spider plot depicting percentage changes in both target tumor burden and tumor markers. Two patients with clinical progression were excluded. Tumor volumes (a) are depicted in same colors as best response of tumor markers (b). Although tumor burden decreased in subjects who achieved partial response (green), tumor marker (CA 125) increased by >30%.

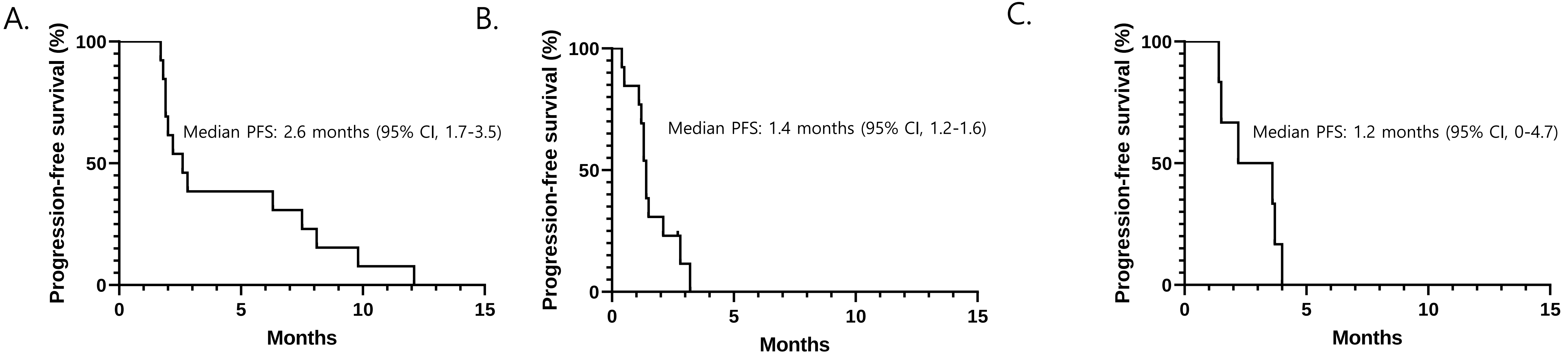

Cancer	Prior to TAS-117 (n=13)	TAS-117 (n=13)	Subsequent line of treatment (n=6)
Breast cancer	3	4	5
Breast cancer	5	6	none
Breast cancer	3	4	none
Breast cancer	4	5	6
Ovarian cancer	3	4	5
Ovarian cancer	2	3	4
Endometrial cancer	3	4	none (expired)
Colon cancer	3	4	none (expired)
Colon cancer	2	3	none (expired)
Rectal cancer	4	5	6
Gastric cancer	8	9	none (expired)
Gallbladder cancer	1	2	3
Non-small cell lung cancer	3	4	none (expired)

Figure S2. Analysis of progression-free survival by A) PFS 1 (prior to TAS 117), B) PFS 2 (TAS 117), and C) PFS 3 (after TAS 117)
Table depicts the line of treatments received.
Abbreviations: PFS, progression-free survival; CI, confidence interval.

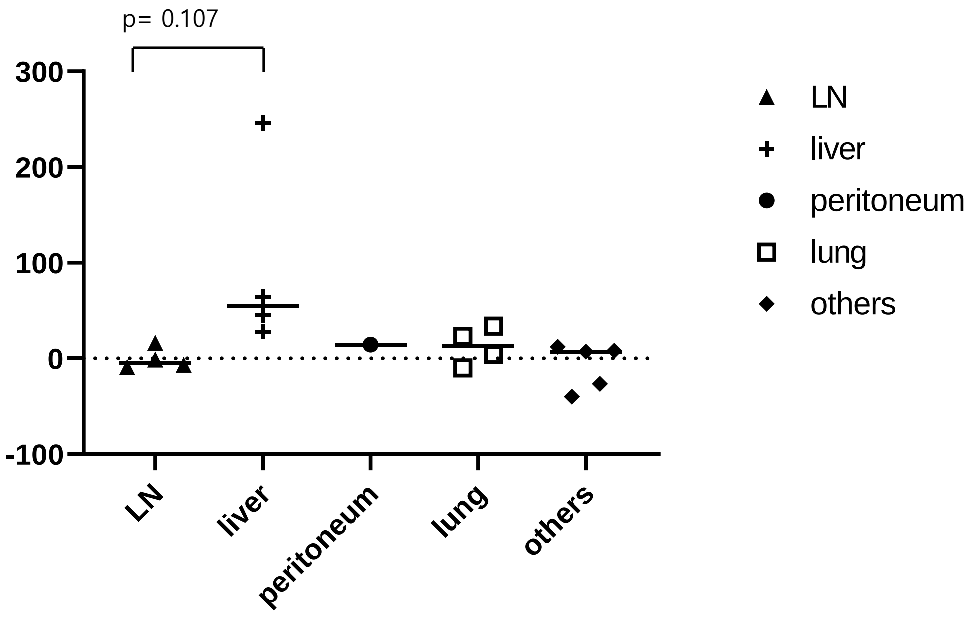

Figure S3. Analysis of responses per metastatic organs by using one-way analysis of variance (ANOVA)
There was no significant difference in progression in all organs (p = 0.076) and the lymph nodes and liver (p = 0.107). Other metastatic organs included: 1) soft tissue around the peri-aortic area, 2) inguinal mass, 3) pelvic cavity mass, 4) seeding nodule in the anterior renal fascia, and 5) spleen.

Abbreviations: LN, lymph nodes.

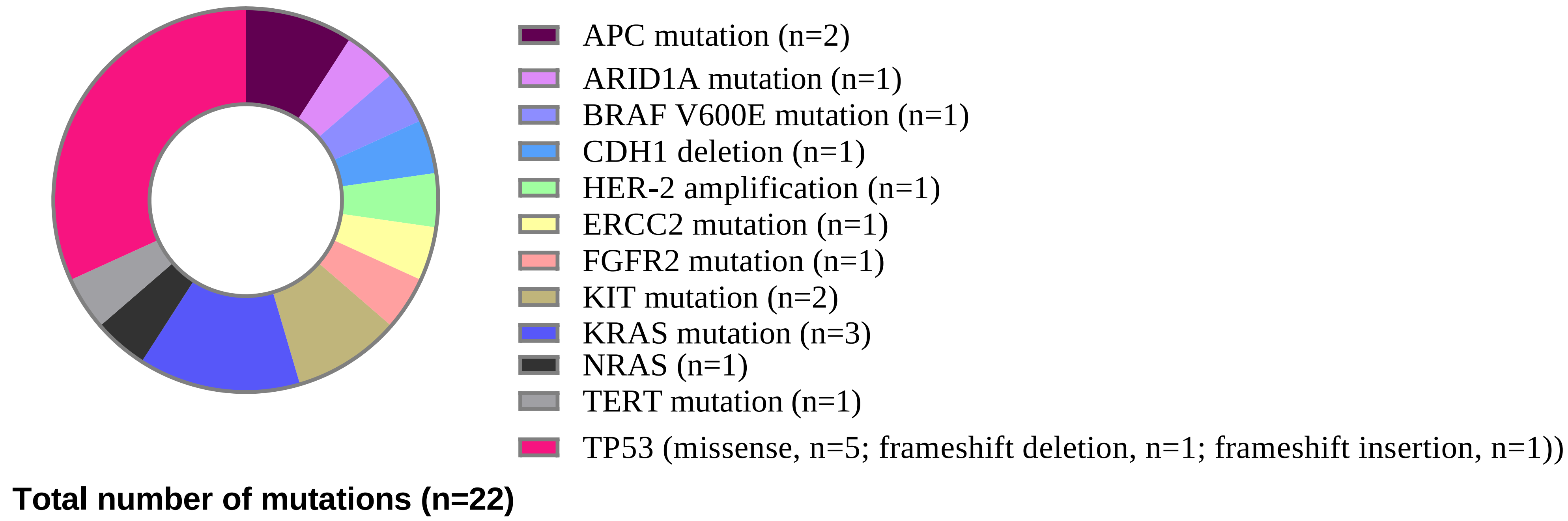

Figure S4. Co-existing mutations identified in patients treated with TAS-117
A total of 22 mutations were identified in 13 patients. TP53 mutations (n = 7) were the most common.

Abbreviations: TP-53, tumor protein 53; APC, adenomatous polyposis coli; KRAS, Kirsten rat sarcoma viral oncogene homolog; ERBB2, Erb-B2 receptor tyrosine kinase 2; CDH1, Cadherin 1; BRAF, B-Raf proto-oncogene; FGFR, fibroblast growth factor receptors; TERT, telomerase reverse transcriptase; ARID-1A, AT-rich interactive domain-containing protein 1A; NRAS, neuroblastoma RAS viral oncogene homolog.

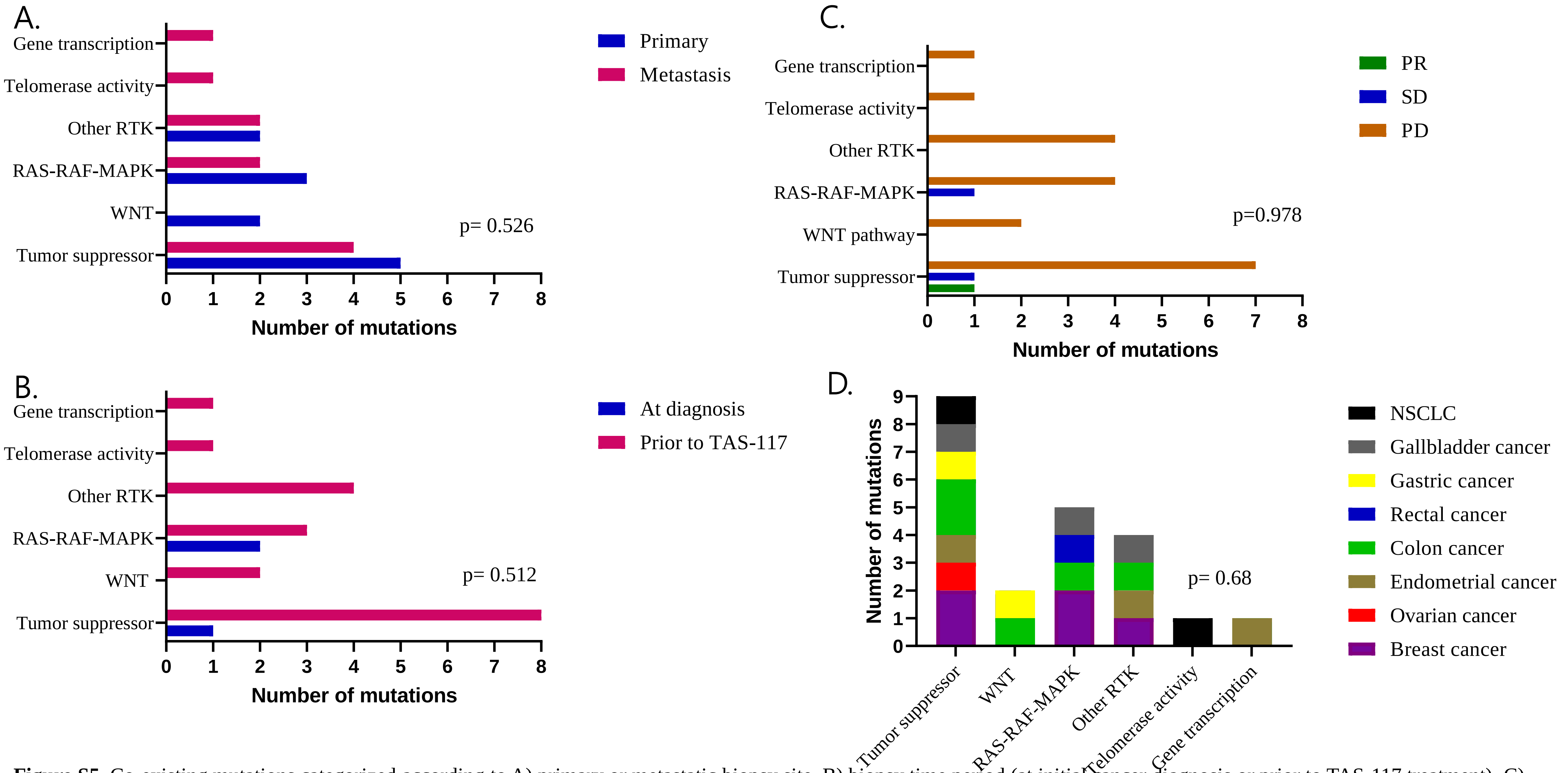

Figure S5. Co-existing mutations categorized according to A) primary or metastatic biopsy site, B) biopsy time period (at initial cancer diagnosis or prior to TAS-117 treatment), C) response evaluation by RECIST criteria (PR, SD, PD), and D) tumor types

There are no correlations between co-existing mutations.

Abbreviations: RTK, receptor tyrosine kinase; RAS-RAF-MAPK, Ras-Raf-Mitogen-activated protein kinase; PR, partial response; SD, stable disease; PD, progressive disease; RECIST, response evaluation criteria in solid tumors; NSCLC, non-small cell lung cancer

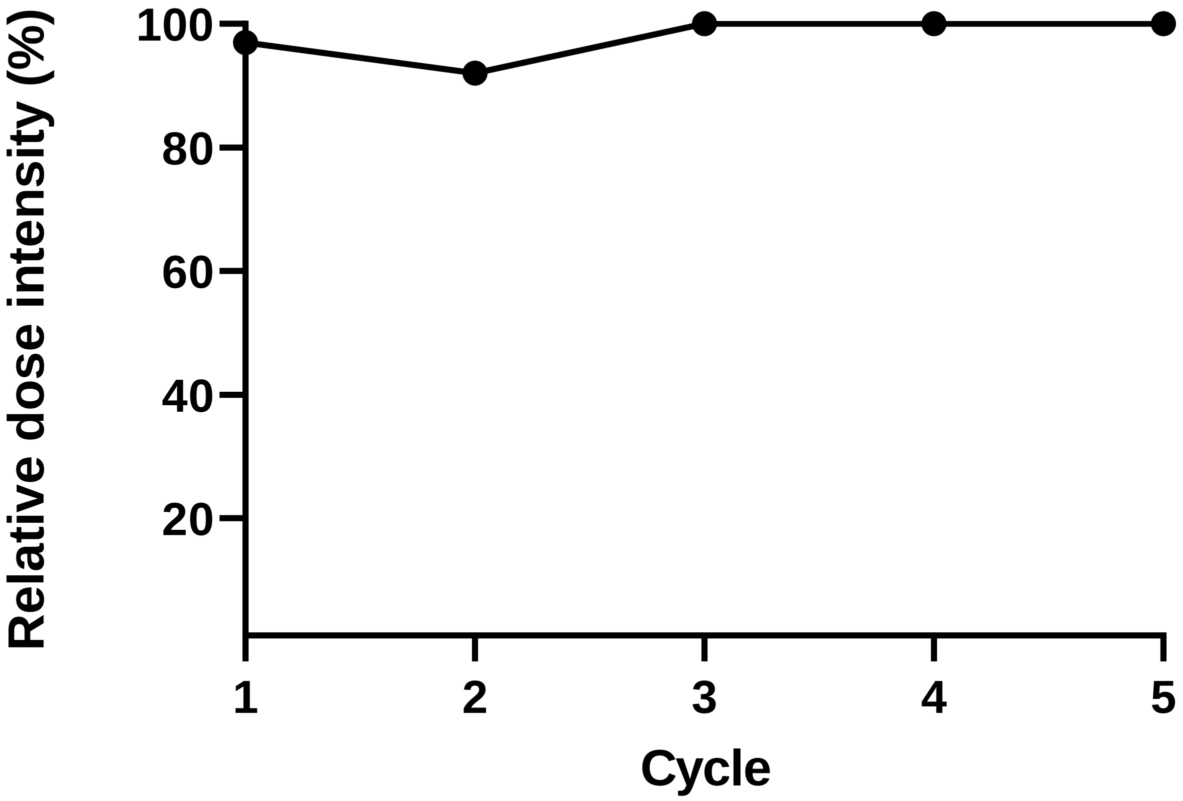

Figure S6. Relative dose intensity of TAS-117

Supplementary Appendix

Investigational New Drugs

Phase 2 study of TAS-117, an allosteric Akt inhibitor in advanced solid tumors harboring phosphatidylinositol 3-kinase/v-akt murine thymoma viral oncogene homolog gene mutations

Jii Bum Lee[1,2], Minkyu Jung[1,2,3], Seung Hoon Beom[1,2], Gun Min Kim[1,2], Hye Ryun Kim[1,2], Hye Jin Choi[1,2], Joo Hyuk Sohn[1,2], Joong Bae Ahn[1,2,3], Sun Young Rha[1,2,3], Hyun Cheol Chung[1,2]

[1]*Division of Medical Oncology, Department of Internal Medicine, Yonsei Cancer Center, Yonsei University College of Medicine, Seoul, Korea*

[2]*Song-Dang Institute for Cancer Research, Yonsei University College of Medicine, Seoul, Korea*

[3]*Brain Korea 21 Plus Project for Medical Sciences, Yonsei University College of Medicine, Seoul, South Korea*

Correspondence: Hyun Cheol Chung, MD, PhD

Division of Medical Oncology, Department of Internal Medicine, Yonsei University College of Medicine, 50-1 Yonsei-ro, Seodaemun-gu, Seoul 03722, Korea

Tel.: 82-2-2228-8132 Fax: 82-2-393-3652 E-mail: unchung8@yuhs.ac

Table of contents

Table S1. Phosphatidylinositol 3-kinase (PI3K)/v-akt murine thymoma viral oncogene homolog (Akt) pathway aberrations of patients

Table S2. Summary of treatment

Table S3. Summary of best response to TAS-117

Table S4. Summary of best response according to cancer types

Table S5. Summary of best response according to PI3K/Akt aberrations

Table S6. Univariate analysis of progression-free survival and overall survival

Table S7. Organs showing disease progression

Table S8. Adverse events during treatment

Table S9. Incidence of adverse events of special interest (AESI)

Figure S1. Spider plot depicting percent changes in both target tumor burden and tumor markers

Figure S2. Analysis of progression-free survival by treatments

Figure S3. Analysis of responses per metastatic organs by using one-way analysis of variance (ANOVA)

Figure S4. Co-existing mutations identified in patients treated with TAS-117

Figure S5. Co-existing mutations categorized by biopsy site, biopsy time period, response evaluation, and tumor types

Figure S6. Relative dose intensity of TAS-117

Table S1. Phosphatidylinositol 3-kinase (PI3K)/v-akt murine thymoma viral oncogene homolog (Akt) pathway aberrations of patients

Cancer type	Line of treatment	NGS time point*	Biopsy site	PIK3CA/AKT aberration	Amino acid change	Other mutations
Breast cancer	4	1	Lymph node	PIK3CA	H1047R	KRAS mutation
Breast cancer	6	2	Liver	PIK3CA	H1047R	HER-2 amplification
Breast cancer	4	1	Chest wall	PIK3CA	H1047R	TP53 mutation, KRAS mutation
Breast cancer	5	2	Orbit	AKT1	E17K	CDH1 deletion
Ovarian cancer	4	2	Ovary	PIK3CA	E545K	ARID1A mutation
Ovarian cancer	3	2	Ovary	PIK3CA	E545K	TP53 mutation
Endometrial cancer	4	2	Ovary	PIK3CA	H1047R	TP53 mutation, KIT mutation, ERCC2 mutation
Colon cancer	4	2	Colon	PIK3CA	Q546K	APC mutation, TP53 mutation, TP53 deletion
Colon cancer	3	2	Colon	PIK3CA	E545A	BRAF mutation, KIT mutation
Rectal cancer	5	2	Rectum	PIK3CA	E545K	NRAS mutation
Gastric cancer	9	2	Stomach	PIK3CA	E545K	TP53 insertion, APC mutation
Gallbladder cancer	2	2	Gallbladder	PIK3CA	E542K	KRAS mutation, FGFR2 mutation
NSCLC	4	2	Lymph node	PIK3CA	E542K	TP53 mutation, TERT mutation

Abbreviations: PI3K/AKT, phosphatidylinositol 3-kinase/protein kinase B; PIK3CA, phosphatidylinositol 3-kinase catalytic subunit alpha; NSCLC, non-small cell lung cancer, TP-53, tumor protein 53; APC, adenomatous polyposis coli; KRAS, Kirsten rat sarcoma viral oncogene homolog; ERBB2, Erb-B2 receptor tyrosine kinase 2; CDH1, Cadherin 1; ERCC, excision repair cross-complementing; BRAF, B-Raf proto-oncogene; FGFR, fibroblast growth factor receptor; TERT, telomerase reverse transcriptase; ARID-1A, AT-rich interactive domain-containing protein 1A; NRAS, neuroblastoma RAS viral oncogene homolog.

*1, At initial cancer diagnosis; 2, prior to TAS-117

Table S2. Summary of treatment

Median duration of treatment (range, months)	1.4 (0.4–3.2)
Median cycle of treatment (range)	2 (1–5)
Dose delays	2 (15%)
Dose reduction	0 (0%)
Dose intensity median (range)	100% (56%–100%)
Subsequent treatment	
Yes	6 (46%)
No	7 (54%)
Subsequent regimen	
Cytotoxic chemotherapy [a)]	3 (23%)
Immunotherapy [b)]	2 (15%)
Targeted agent [c)]	1 (8%)

[a)] Patients were treated with 1) pegylated liposomal doxorubicin, 2) capecitabine, and 3) belotecan and cisplatin.

[b)] Patient was enrolled in clinical trial and was treated with PD-L1 and IDO inhibitor.

[c)] Patient was treated with regorafenib.

TABLE S3. Summary of best response to TAS-117

CR	0 (0%)
PR	1 (8%)
SD	2 (15%)
PD	10 (77%)
Overall response rate (CR+PR)	1 (8%)
Disease control rate (CR+PR+SD)	3 (23%)

Abbreviations: CR, complete response; PR, partial response; SD, stable disease; PD, progressive disease

Table S4. Summary of best response according to cancer types

**Cancer**	PR	SD	PD
Breast cancer (n=4)	0	2	2
Ovarian cancer (n=2)	1	0	1
Endometrial cancer (n=1)	0	0	1
Colon cancer (n=2)	0	0	2
Rectal cancer (n=1)	0	0	1
Gastric cancer (n=1)	0	0	1
Gallbladder cancer (n=1)	0	0	1
NSCLC (n=1)	0	0	1

Abbreviations: n, number; CR, complete response; PR, partial response; SD, stable disease; PD, progressive disease; NSCLC, non-small cell lung cancer

Table S5. Summary of best response according to PI3K/Akt aberrations

**PI3K/AKT aberrations**	PR	SD	PD
PIK3CA mutation			
E542K (n=2)	0	0	2
E545A (n=1)	0	0	1
E545K (n=4)	1	0	3
H1047R (n=4)	0	1	3
Q546K (n=1)	0	0	1
AKT1 mutation			
E17K (n=1)	0	1	0

Abbreviations: n, number; PI3K/AKT, phosphatidylinositol 3-kinase/protein kinase B; PIK3CA, phosphatidylinositol 3-kinase catalytic subunit alpha; CR, complete response; PR, partial response; SD, stable disease; PD, progressive disease;

Percentages may not sum to 100 because of rounding.

Table S6. Univariate analysis of progression-free survival and overall survival

Variables	Total number of patients (%)	PFS (months)	p-value	OS (months)	p-value
Line of treatment for TAS-117 (<4 or ≥4)	3 (23%) vs. 10 (77%)	1.3 vs. 1.4	0.575	2.9 vs. 6.9	0.743
Royal Marsden Score (0,1 vs. ≥2)	6 (46%) vs. 7 (54%)	1.3 vs. 2.1	0.219	4.3 vs. 9.2	0.139
Grim-Score (0,1 vs. ≥2)	6 (46%) vs. 7 (54%)	1.3 vs. 2.1	0.219	4.3 vs. 9.2	0.139
Number of metastatic (<2 vs. ≥2)	1 (8%) vs. 12 (92%)	1.3 vs. 2.1	0.594	4.8 vs. 10.1	0.391

Abbreviations: GRIm-Score, Gustave Roussy Immune Score; PFS, progression-free survival; OS, overall survival

Table S7. Organs showing disease progression

Metastasis	Number (%)
**New site of metastasis**	**1 (9%)**
Liver¶	1 (9%)
**Progression of target lesions**	**9 (82%)**
Lung	2 (18%)
Lymph node*	1 (9%)
Peritoneum	1 (9%)
Liver, peritoneum	1 (9%)
Lung, liver, peritoneum	2 (18%)
Lung, liver, bone	1 (9%)
Large intestine, peritoneal carcinomatosis¶	1 (9%)
**New site of metastasis (A) with progression of target lesion (B)**	**1 (9%)**
Common bile duct and pancreas (A), lung and lymph node (B)	1 (9%)

Organs of progression are categorized as newly developed lesion or progression of previous metastatic lesions.

An ovarian cancer patient with partial response progressed with liver metastasis (new site of metastasis). Two breast cancer patients with stable disease progressed to lymph node (target lesion) and large intestine and peritoneal carcinomatosis, respectively.

*Ovarian cancer with partial response. ¶Breast cancer patients with stable disease

Table S8. Adverse events during treatment

Adverse event	Grade 1	Grade 2	Grade 3	Grade 4	All grades
**Any event**	8 (24%)	22 (65%)	3 (9%)	1 (2%)	34 (100%)
Anorexia	1 (9%)	2 (18%)	1 (9%)	0 (%)	4 (36%)
Constipation	0 (%)	1 (9%)	0 (%)	0 (%)	1 (9%)
Diarrhea	1 (9%)	1 (9%)	0 (%)	0 (%)	2 (18%)
Nausea	2 (18%)	1 (9%)	0 (%)	0 (%)	3 (27%)
Mucositis	1 (9%)	0 (%)	0 (%)	0 (%)	1 (9%)
Skin rash	2 (18%)	3 (27%)	0 (%)	0 (%)	5 (45%)
Itching	0 (%)	2 (18%)	0 (%)	0 (%)	2 (18%)
Fatigue	0 (%)	1 (9%)	0 (%)	0 (%)	1 (9%)
Headache	0 (%)	2 (18%)	0 (%)	0 (%)	2 (18%)
Back pain	0 (%)	1 (9%)	0 (%)	0 (%)	1 (9%)
Shoulder pain	0 (%)	2 (18%)	0 (%)	0 (%)	2 (18%)
Dyspnea	0 (%)	1 (9%)	0 (%)	0 (%)	1 (9%)
Pneumonia	0 (%)	1 (9%)	0 (%)	0 (%)	1 (9%)
Pulmonary thromboembolism	0 (%)	2 (18%)	0 (%)	0 (%)	2 (18%)
Hyponatremia	0 (%)	0 (%)	1 (9%)	0 (%)	1 (9%)
Hyperglycemia	1 (9%)	1 (9%)	1 (9%)	1(9%)	4 (36%)
Neutropenia	0 (%)	1 (9%)	0 (%)	0 (%)	1 (9%)

Adverse events were those with onset after enrollment to last follow-up after disease progression.

Table S9. Incidence of adverse event of special interest*

AESI	Grade 1	Grade 2	Grade 3	Grade 4	All grades
**Any event**	4 (36%)	5 (45%)	1 (9%)	1 (9%)	11 (100%)
Mucositis	1 (9%)	0 (0%)	0 (0%)	0 (0%)	1 (9%)
Skin rash	2 (18%)	2 (18%)	0 (0%)	0 (0%)	4 (36%)
Hyperglycemia	1 (9%)	2 (18%)	1 (9%)	1 (9%)	5 (45%)
Neutropenia	0 (0%)	1 (9%)	0 (0%)	0 (0%)	1 (9%)

Abbreviations: AESI, adverse event of special interest

Adverse events were those with onset after enrollment to last follow-up after disease progression.

Percentages may not sum to 100 because of rounding.
